# Strengths and weaknesses of food eco-labeling: a review

**DOI:** 10.3389/fnut.2024.1381135

**Published:** 2024-03-27

**Authors:** Ornella Tiboni-Oschilewski, Magdalena Abarca, Fabiana Santa Rosa Pierre, Alice Rosi, Beatrice Biasini, Davide Menozzi, Francesca Scazzina

**Affiliations:** ^1^Department of Food and Drug, University of Parma, Parma, Italy; ^2^Independent Researcher, Santiago, Chile

**Keywords:** food choice, eco-label, food labeling, front-of-package labeling, sustainable foods, food environment

## Abstract

Food labeling is increasingly expanding and adding more information to the food package. There is strong evidence about nutrition labeling effectiveness in driving food choice, especially if displayed in the front of package (FoP). Despite the growing attention to nutrition and sustainable diets, few countries have implemented sustainable labels or *eco-labels* that could address economic, social and/or environmental concerns. Implementing new techniques of eco-labeling emerges as a consumer-focused solution. However, evidence of the effectiveness of eco-labeling in driving consumers’ choices is heterogeneous and not univocal. Thus, this review aims to summarize the evidence about the effectiveness of FoP eco-labeling in driving food choice and provide a reference framework of the eco-labeling initiatives relative to food package labeling. This narrative review addresses both the potential benefits as well as the main concerns that arise from the use of eco-labels. Although eco-labeling seems to provide a series of sustainability benefits for producers and consumers, the implementation of such policies should take into consideration potential trade-offs and inter-sectorial coordination to obtain bigger impacts, assuming that a policy itself cannot transform the whole food system. Eco-labeling could be encouraged and implemented within a set of policies shaping sustainable food systems.

## 1 Introduction

Malnutrition in all its forms and climate change are intimately related. Food systems are one of the main causes of climate change being responsible for 21–37% of greenhouse gas emissions, and yet food systems are deeply affected by the consequences of climate change (1, 2). Sustainable food systems seem to be one tool to address many challenges, even economic and social inequity (3). However, due to the broad dimension of food systems, a virtuous transformation requires policies directed to different actors of the food system and should be implemented and aligned in a holistic and integrative umbrella. Food environments, which refer to physical, economic, sociocultural and policy framework, shape food accessibility, affordability, safety and ultimately food preferences. In order to deliver qualitative information to consumers, food labeling is a key food environment component to promote safer and more accessible alternatives (4). Food labeling is not only one of the recommendations of the World Food Security Committee Voluntary Guidelines on Food Systems and Nutrition (4), but it also contributes to consumer rights, particularly consumer’s right to know, which allows an informed food choice.

Product labeling, and specifically food labeling, corrects information asymmetries that exist along the value chain, especially between producers and consumers. The inability of consumers to evaluate certain quality features before or even after purchasing, such as production methods, increases the importance of credence attributes. These characteristics are then transformed into search attributes, often in the form of labels (5, 6). In this sense, food have labels that reveal information considered relevant by the regulatory entity and fulfills a democratic function, as it is fair that consumers have clear and understandable information on the products they are acquiring and consuming (7). Despite the extensive application of the nutrition labeling (8), weather voluntary or mandatory, its usefulness is especially recognized when combined with other policies that contribute to the same result, instead of isolated policies that only include labeling (1, 9), e.g., front of package labeling (FoP) plus healthy food subsidies.

In addition to consumer rights, food labeling fulfills a practical function: it has been shown that having such information promotes a change in consumer behavior when buying (7). Secondly, labeling policies often aim to ensure and stimulate a fair competition among companies on the market (10). However, it is important to bear in mind that the final objective of official labeling is to encourage informed consumption on different aspects of the product, weather nutritional, and environmental labeling. Thus, when evaluating the effect of a new label, it is necessary to ask whether the number of informed consumers has increased, whether there has been a change in consumption patterns due to the greater number of informed consumers, and whether said change has contributed to achieve the proposed social and/or environmental objective (10). The main limit of food labels is related with the imperfect recall by consumers, and their possible confusion between positive and negative characteristics, when the information given is technical or complex (6).

According to ISO (11), there are three types of environmental labels: Type I are those with clearly defined criteria (excludes food and beverages), Type II are self-declared claims with no criteria nor schemes, and Type III declarations using a life-cycle approach. However, these specifications are not intended for certifications or registrations. Eco-labels identify those products compliant with specific environmental performance criteria and can be owned or managed at government level, non-profit organizations or by the private sector. To enable more informed and sustainable food choices, the European Commission has expressed its commitment to develop a legislative framework for sustainable food systems, where sustainability labeling would be a central element, encompassing nutritional, environmental and social pillars associated to food (12).

So far, most of the evidence of eco-labeling is focused on the agriculture and food sector, since these are the sectors that contain the greatest number of eco-labels (13, 14). Moreover, to date, eco-labels and its research are more prevalent in developed countries, while a few very low income countries apply this policy (14). In Europe, the use of sustainability claims and labels is growing, as demonstrated by a 2.83% annually increase from 2005 to 2021 in the average share of such claims and labels on newly introduced products. For example, France introduced the environmental labeling of food in 2020, to become mandatory within 5 years (15). Specifically referring to the products bearing environmental-oriented claims and labels, those accounts for around 68.2% of the overall trend (16).

Eco-labels can be single-attribute, when focusing on a specific lifecycle stage or a specific environmental impact of a product/service, or multi-attribute, if they focus on the whole lifecycle or multiple environmental impacts associated to a product/service (17), being the latter the least used format (18). Accordingly, when applied to food, they do not always refer to the whole product, but can also refer to one specific aspect of the product, such as the production methods (e.g., organic labeling), the environmental impact(s) measured through the life cycle assessment (e.g., carbon footprint labels), or the package itself (e.g., recyclability), among others. Evidence of its effectiveness in driving consumers’ choices is hard to systematically evaluate, as there are too many different types and requisites. The number of eco-labels has increased over time reaching a number of 456 eco-labels in a total of 199 countries (19).

Considering the great diversity and non-standardized format among industries and countries, eco-labeling in the food industry is an increasingly debated topic. Current eco-label implementation guidelines are not open-access (ISO) nor specific for food products (20), which does not facilitate the regulation process. Mostly, guidance on how to obtain a certain certification can be found (21), rather than guidance on the regulation and implementation process. Policymakers could benefit from evidence-based guidance, considering that it has become a hot topic in food regulations. Consumers and manufacturers could also benefit from a standardized eco-label and a transparent implementation process. This guidance could be even more important for the global south, as this is a new debate and not many resources nor technical guidance is available. Specifically, which characteristics, by whom and upon which indicators this certification could be given are important topics to elucidate. Accordingly, the objective of this study is to provide a narrative review to summarize the evidence about the effectiveness and pros and cons of FoP eco-labeling in driving food choice to generate a series of recommendations for its effective implementation in public policies.

## 2 Materials and methods

Following Tranfield et al. (22), this review was developed by initially defining the framework and the keywords to be used in the search, such as (food AND ecolabel), (food AND ecolabeling), and [food AND (sustainability OR sustainable OR environmental) AND (labeling OR label)]. The literature review was carried out by the first author during June 2023 through PubMed, Scielo, Epistemonikos, Web of Science and Cochrane. The keyword search was focused on title and abstract. If the keywords were present, the abstract screening was first performed, followed by the main text. Articles were selected if related to eco-labels applied to food, with eco-labels referring directly to food item(s) or to the packaging. Manuscripts discussing eco-labels not applied to food or not specifically referring to food item(s) or its/their packaging were therefore excluded. Also, only packaged food has been considered, thus canteen or restaurant menus were excluded. Only systematic reviews, reviews and original research articles were retrieved. No timeframe for the search was used, as most studies date 2000 onwards. Both real and virtual experimental settings involving the general population or certain population subgroups (e.g., children) were included. Existing and non-existing, i.e., real (e.g., supermarkets) or hypothetical contexts (e.g., laboratory experiments), as well as evaluative (e.g., traffic-light colors or presence of a logo) and/or descriptive (e.g., quantitative environmental values) eco-labels were considered. Language restrictions were not necessary because all the identified articles were in English or Spanish, languages that the authors are proficient in. Finally, other papers not retrieved from the application of the keywords in the above mentioned databases were considered due to their relevance to the topic of food labeling (23–25).

## 3 Results and discussion

A total of 58 records were reviewed, most of which original articles (*n* = 49), followed by systematic reviews (*n* = 3), systematic reviews and meta-analysis (*n* = 2), reviews (*n* = 2), meta-analysis (*n* = 1), and narrative review (*n* = 1). Of the original studies included in this review that explicitly stated country of study, United Kingdom was the most studied one (*n* = 7), followed by the United States (*n* = 6), China (*n* = 3), France (*n* = 3), Germany (*n* = 3), and Italy (*n* = 3). Most of the studies were focused on Europe (*n* = 33). Supplementary Table 1 provides an overview of the collected literature including information on authors, type of the study, the study design, eco-label content/claim/image, and main outcomes.

The results are organized according to five different areas of impact or concern when designing eco-labeling policies (Table 1): change in consumer’s perception, intention or behavior (*n* = 21); price and value relationship between food products and eco-labeling (*n* = 16); producer’s perspective (*n* = 6); institutional framework and label development (*n* = 13); and eco-label design and excessive information (*n* = 14). Among these, some of the collected records fall under multiple areas (*n* = 11).

**Table 1:** Description of the topics in which the collected literature is organized.

Topic	Description	References
Change in consumer’s perception, intention or behavior	It collects evidence about the impact of eco-labels on consumer’s sustainable purchases and the influence they have on the attributes considered at the time of choice. Examples of long-term effect of such labels are included.	*N* = 21 (26, 27, 28, 29, 30, 31, 32, 33, 34, 35, 36, 37, 38, 39, 40, 41, 42, 43, 44, 45, 46)
Price and value relationship between food products and eco-labeling	It considers the price variation of food products bearing eco-labels and its potential implications from the producers’ and consumers’ perspective, including the willingness to pay. Cross-cultural differences are discussed.	*N* = 16 (10, 14, 24, 47, 48, 49, 50, 51, 52, 53, 54, 55, 56, 57, 58, 59)
Producer’s perspective	It values the involvement of producers in the transformative framework moving toward more sustainable, accessible, and affordable standards of production and consumptions. Both positive and unintended outcomes are discussed.	*N* = 6 (10, 39, 60, 61, 62, 63)
Institutional framework and label development	It discusses the importance of the role of certifying entities and the interaction among stakeholders in eco-label implementation. Trust and understanding of the labels are particularly considered.	*N* = 13 (10, 14, 23, 46, 48, 64, 65, 66, 67, 68, 69, 70, 71)
Eco-label design and excessive information	It refers to the influence and/or effectiveness of certificate or eco-labels design in risk perception and behavior change in consumers. The effect of the presence of multiple labels and socio-demographic characteristics are discussed.	*N* = 14 (25, 26, 41, 42, 53, 59, 67, 72, 73, 74, 76, 77, 78, 79)

Some references are mentioned under more than one topic, therefore the number of references reported here overcomes the number of records reported in the main text (*n* = 58).

### 3.1 Change in consumer’s perception, intention, or behavior

As to whether or not the presence of an eco-label influences consumers’ sustainable purchases and their impacts, discordant results can be observed. On one hand, a pilot study, conducted in an online supermarket from van der Waal et al. (26), found that an explanatory health claim added to an explanatory sustainability claim was not effective in fostering sustainable purchases, while explanatory sustainability claim alone was even associated to lower correspondent purchases from people having low environmental attitudes. Whereas van Bussel et al. (27) found in their systematic review that price, taste, and individual health were more important for consumers than sustainability.

Nonetheless, positive results were instead found in other studies (28–35) in which the application of eco-labels changed consumer behavior, proving that eco-labels can influence the number of attributes considered when purchasing a product. More specifically, when different wordings are compared, those referring to “sustainably” or “locally” sourced products were chosen the most (32) along with “organic,” as stated in a systematic review (36), similarly to those with traffic lights (37). Eco-labels could also lead to an increased perceived quality of the product (38). Regarding other perceptions, consumers do not have a negative perception of the brands in the presence of an eco-label (39).

When buying or acquiring foods, a clear informative seal that provides easy-to-understand information (40) could trigger a choice toward more sustainable products, even for people who do not have prior information on sustainability (41), as this choice could also be partly subconscious (42). However, it must be taken into consideration that there are substantial differences among preferences between different countries, the motivations of purchase and consumers themselves, such as demographic characteristics and human and cultural values (e.g., if it is a desired attribute; if it is accepted) (43). For example, among the reasons of choosing a label, Zepeda et al. (43) found that the object itself (such as the type of label, quality, and hedonic components) was the main reason (64.4%), followed by the label source (such as the credibility, reputation) (45.5%), and then the consumer characteristics (e.g., willingness to pay, attitude toward the label, etc.) (42.1%).

Although some authors found different results (44), the tendency of the evidence is that this impact in changing consumer behavior is best observed with those who already show some degree of concern and knowledge about the impact of their consumption patterns on the environment (45), or for those who understand the eco-label clearly, as this was found to be a prerequisite of using the eco-labels (46). The sustainability knowledge plus the eco-label acted as a consistent predictor of consumers’ choice strategy (32).

Eco-labeling could also have long-term impacts over consumers. For instance, in the United States, a change in consumption behavior was seen with the introduction of this new category of information on food products. One of the most visible examples was in 1990 with the introduction of the “dolphin-safe” label on tuna cans. The market share of canned tuna companies certified as “protectors of dolphins” had a constant growth reaching more than 90% of the market share, an effect that was maintained over time (28). This accounts for a long-term impact that labels can have on the various market players in terms of sustainability.

### 3.2 Price and value relationship between food products and eco-labeling

One effect that certification of meeting a sustainability standard can produce is that producers increase the price of said product, either because it effectively makes the production of that good more expensive, or because of the possibility of classifying said good as “premium” in relation to other similar ones that do not have the certification. However, a studied carried out in Japan and the United Kingdom found no distortionary impact on consumer preferences for eco-labels (47).

Producers may invest in innovation to meet the new standards and thus become certified. For example, in the case of the “dolphin safe” label, it was plausible that the price of tuna would increase as a result of the new production techniques, and this would particularly affect the lowest income households, which may not be willing or have the resources to pay for the “dolphin safe” products, which could imply a redistribution of welfare from low-income households to high-income households (10). If standards are so strict that production costs exceed the prices consumers are willing to pay, consumers will seek products with lower standards, so producers will not continue innovation efforts, making such high standards lose meaning.

Regarding consumer’s willingness to pay for a labeled-product, heterogenous results have been obtained, as some studies found no intention, or intention only if accompanied with discounts, to a higher price for them (48). On the contrary, both a meta-analysis (49) and original articles (50–53) found that consumers were willing to pay a premium price, even though the eco-product was not the preferred one according to taste (54). This could be due to the hypothetical scenarios, where no real money is on the line, as it has been reported that consumers overestimate the willingness to pay on this type of studies (55).

Higher prices may be the case for organic labels, as confirmed by meta-analyses and/or systematic reviews (24, 49, 56). The willingness to pay seems to be more associated with higher label penetration into market than to the certification transparency itself (57). However, familiarity with the label seems not to be the case for China, as Zhu et al. (58) found that Chinese consumers’ willingness to pay a higher price was only if the Chinese organic label was accompanied with one from abroad (e.g., EU organic). However, it is important to say that a study found that consumers were willing to pay up to 23% higher prices for familiar tagged (unverified claims) products compared to an existing certified eco-label such as Marine Stewardship Council (MSC) (59).

Finally, cross-cultural differences are also an interesting point to take into account. For example, one study compared the same products between German and Canadian consumers (48). The authors found that German consumers prefer more sustainable products than Canadians, influencing also the willingness to pay. In general, richer and more populated countries are prone to consider environmental issues, however, there are some very low-income countries also concerned about sustainability (14). Therefore, cultural preferences also influence the willingness to pay and the importance level of the sustainability attribute of food.

### 3.3 Producer’s perspective

It is important to consider that transformative effects are reached if eco-labeling is situated in a legal and institutional framework that seeks to move from less sustainable ways of production and consumption toward others that are more sustainable and accessible both physically and economically. A review also suggested that producers must be involved in those changes as it is expected to impact the manufacturing process (60).

A direct effect derived from the implementation of labeling is related to the transformation in product elaboration. As green consumers prefer more sustainable products, there is an incentive for producers to adopt new, more innovative and sustainable practices to satisfy these consumers. This is not only true for big companies, but also for farmers and producers, as they also prefer having an eco-label, however, they were mostly worried about long-term health effects than for increasing exports or other aspects (61). When establishing a type of certificate or eco-label, some producers are forced to reveal negative characteristics of their products. Therefore, it is plausible that producers decide to change the characteristics of their product during the production process to meet the standards of the implemented certificate and not risk losing customers-in the short term-and market share and revenue-in the mid and long term (10).

Likewise, this may lead to more producers wanting to gather information on the environmental characteristics of their products to be more competitive, which could lead to further innovation and more sustainable solutions. This process could become a real challenge when measuring the environmental footprint, which could in turn retract producers of adhering to the label (62). There has been reluctance from retailers to include these type of labels, even though it has not been shown to result in negative consumer side perceptions (39), but in some cases it has not resulted in increased profits for producers either (63).

### 3.4 Institutional framework and label development

Even though eco-labels are more present in wealthier countries (14), the following evidence could be applied for the institutional framework in any country. Regulated and transparent certifying entities could help strengthen labeling through inspections, certifications and fines. As stated in the first section, consumer’s trust in the label is fundamental for behavioral change. When doing so, it is important to consider interaction among stakeholders, as they could be a barrier into this implementation due to lack of agreement (64).

It was suggested that consumers’ acceptance of sustainability could differ by whether consumers trust or distrust the label information (48). Differences between countries could be attributable to trust in the public agencies, thus altering the impact of the label. Labels that are not consistent with achievable standards, inspections, certification services and sanctions could not be effective, and could even generate major market disruptions (10). However, a review suggested this could also be a result of inconsistency in reference values and indicators or dimensions considered; therefore, the same profiling models should be used (65). Even though there are some doubts about the transparency of some certification processes, e.g., of sustainable fisheries, a study found that products certified had between 3 and 5 times less probabilities to have harmful practices (23); lack of credibility (46, 66, 67); unfamiliarity (68); confusion regarding the content of the label since it affects its effectiveness (10). Along with trust in the label (66), being provided with clear and meaningful information on eco-labels is particularly relevant for consumers as they may misunderstand it (69, 70). According to the authors’ explanation, the importance of organic food to consumers could be partially explained by higher understanding of consumers to this label in comparison to others (71). This is further corroborated by Kaczorowska et al. (68), who suggested that unfamiliar logos have less probability of being used. In general, a higher understanding of sustainability standards leads to a more frequent use of the labels for consumers’ decisions. Thus, ease of understanding is a prerequisite for the functionality of any label. One possibility is doing self-explanatory labels as these reach higher understanding among consumers (71). In addition, educational campaigns that allow the general public to correctly understand eco-labeling, its meaning, its objectives and its relevance to the environment (70) are required. Failure to do so could damage consumer confidence in the system. If the education campaigns about the new certificate are appropriate and widely disseminated, a greater impact can be generated.

### 3.5 Eco-label design and excessive information

The design of the certificate or eco-label is key for it to produce the expected effects, both for consumers and for the whole system. Simple and clear graphic designs are more effective in influencing the risk perception and consumer behavior changes than any information table with text or numbers (72, 73). However, a recent systematic review and meta-analysis concluded that despite different eco-label formats, all lead to selection and purchase of more sustainable products (74).

It is also necessary to take into account the influence produced by the colors of the labels as they can lead consumers to be confused, as it is understood that products with a green label are healthier and more sustainable (75). In fact, a recent review found that there was more effectiveness in traffic-light systems when they showed an “alert” (e.g., red color) rather than the green one on purchase intention or purchase behavior (73).

Another relevant aspect is the location of the stamp, certificate, or information. The evidence shows that the frontal and central location of the container or package produces better impacts and, in short, causes a greater reduction of the environmental impact because consumers choose more sustainable products (73). This position could lower the unawareness risk, as this is also a concern. For example, in a Greek study up to 26% of the participants did not notice the content of the label (53). Even worse results were found in Italy, where up to 75% of consumers did not notice the label (76). Therefore, educational policies are necessary for the label to be understood and noticed (76).

As there is an ecosystem of labels on foods, it is a common suggestion having different categories of labels together, for example, nutritional and environmental. But this suggestion has discrepancy on its impacts over the consumer (26, 72), as it induces trade-offs between both (73). In addition, consumers have been found to limit cognitive ability, and to be distracted or dissuaded from considering many labels (41), and thus losing effect of the label (42). For example, a recent study in France showed that there is a decrease in the impact of adding more scores (25). However, evidence also shows that consumers tend to look for labels that have value to them, therefore having more labels do not necessarily mean a higher value for all consumers (77).

It was found in Germany that consumers even preferred a combination of labels, as one label did not dimmish the effect of the other, and consumers handled both labels being able to face contradictory information and trade-offs (67). For example, having two different indicators allows this differentiation to be made (78) and also to compare them (78). This is especially relevant when taking into consideration that the familiarity and trust are core to the label’s impact, and that consumers tend to be skeptical about unfamiliar and vague labels (77).

To establish a robust labeling or certification system, it is essential to choose between a binary seal (i.e., the product either meets the standard or not) or a label that considers a gradual score (i.e., allowing for gradation and differentiation by percentages). For example, a study conducted in the United States showed that an eco-label or certification is less useful and had lower importance score than a sustainability tag (in the study defined as a word, phrase or simple picture either on the promotion or in the product itself) (59). Thus, simplicity could be even more important than the process of certification itself.

Finally, socio-demographic characteristics, such as gender, age, and educational level, have been found to differently affect expectations regarding the information related to environmental sustainability, as investigated for food packaging by Chirilli et al. (79). For example, women are more environmentally conscious, while younger and more educated consumers are more aware of labels and environmental sustainability, respectively, than their counterparts (79).

## 4 Policy recommendations and conclusions

Many elements that should be analyzed when planning policies on eco-labels. An important aspect to consider regarding the evidence today is that most of the data on customer behavior has been obtained via online questionnaires and experimental settings. Therefore, these results should be carefully interpreted, especially regarding consumer’s willingness to pay. There remains a gap in the evidence when considering point-of-sale decisions and how real-world dynamics affect customer’s decisions.

According to our results, these policies should have specific objectives and measurements indicators to evaluate their impact. Monitoring and evaluation should be made accordingly to new consumer’s interests and values.

**Figure 1:**
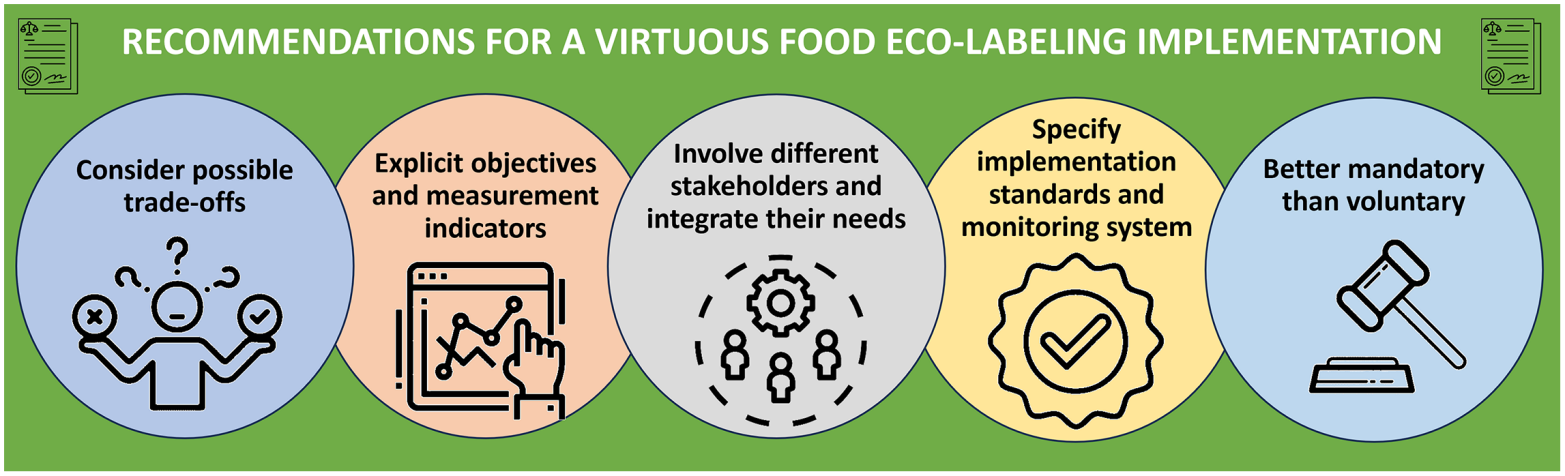
A synthesis of the recommendations to implement eco-labels. Created with Canva.com.

The recommendations that arise from the collected evidence are the following and are synthetize in Figure 1:

- Eco-label can drive an intention and/or behavior change in consumers when noticed and well-interpreted. The application of clear, simple and informative labels is crucial for its effectiveness. Higher impact could be achieved through education campaigns that communicate not only about the label itself, but also the reason for its application and its potential impacts by empowering consumers.
- Policymakers should organize discussion opportunities with different stakeholders, including the private sector. If the latter is not involved and committed, it could be a huge risk of retracting and delaying the label implementation. If involved, it could add much value to the policy itself and its impacts. The power of the private sector not only relies in the production process transformation, but also in education and by performing sustainability assessments.
- The institutional framework under which the products are certified and controlled to ensure the right of bearing the labels must be clear, transparent, and properly communicated. Avoidance to vague terms such as “sustainable” or “natural” should be stated. Its indicators and scope must be specified and defined in concrete terms. A clear methodology and indicators and even cut-off points should be addressed. These aspects are of most importance to allow consumers’ trust on the label. The Government and external controlling entities can be involved. Regulation and certification by a governmental institution or an autonomous and independent third party is an asset. The education campaigns could add this information in its content.
- Colors and location should also be specified, as well as the words to be used. Location is recommended to be at the front.
- Trade-offs between simplicity and integration between other labels should be analyzed locally and discussed with different stakeholders. Whether it is one label that involves more than one aspect or different labels regarding different aspects should be assessed against consumer’s priorities and national priorities. The label must allow consumers to easily compare products. However, it is preferable to have an imperfect-designed label than none.
- The price is an aspect that must be discussed when designing these policies. Even though there are many consumers willing to pay more, vulnerable populations may be left behind. Innovation and technology should be analyzed to even the expenses. It could be discussed that, if even a premium is imposed by some companies, it would still be environmentally beneficial to have a different choice only for some consumers. Also, regulations that impose mandatory labeling and do not include clear standards, tests to verify adherence to the required standards, or a transparent certification and an effective compliance system, leads to confusion and higher costs.
- Ideally, this kind of policies should be mandatory, especially when they show negative attributes of the product. However, in doing so, adequate support to small and medium enterprises should be discussed and eventually provided to be fair and prevent disparities.

Eco-labels should be implemented in combination with regulatory tools such as banning some products or processes and applying fiscal measures (13). This type of labels could also be applied in other contexts, such as food canteens at work (80), as it has been found to lead to a more sustainable menu selection (35). However, it is important to consider that there is evidence that the price of eco-labeled products is the main factor that limits the purchases of said products that are certified and this, affects more vulnerable households (13). A limitation of this study is that no quantitative data are estimated. Also, our results on country of origin of the research are biased, as this is not a systematic review, and it may fail to provide an accurate picture based on the available literature on this topic. Therefore, future studies should aim to systematically review the broader effects of eco-labels and propose strategies in specific real-life settings for overcoming the challenges to extending eco-labeling to all economic and social sectors, considering factors like eco-labeling implementation costs, the culture, and social features.

Even though the effectiveness of the impact of eco-labels on climate change requires further investigations, labeling certifications and its measurement, as well as raising public awareness is essential to move toward a more sustainable food system and achieve the Sustainable Development Goal #12, sustainable consumption and production.

In conclusion, to our knowledge, this is the first study to review the strengths and weakness of the eco-labeling in food products offering specific recommendations for policymakers when planning and implementing eco-labels. One of the biggest challenges for researchers and policymakers is to look for the best way to make eco-labels visible, accessible and useful to all consumers across different economic sectors, especially those who are facing food insecurity and malnutrition, and not only to the “greener customers.” A combination of many policies rather than relying on a single one may be the solution for a more sustainable food system and food alternatives for consumers. Indeed, one single policy or measure by itself will not make the food transformation that is urgently needed, but a combination of many policies.

## Author contributions

OT-O: Conceptualization, Data curation, Formal Analysis, Methodology, Project administration, Writing – original draft, Writing – review & editing. MA: Conceptualization, Data curation, Formal Analysis, Methodology, Writing – original draft. FaS: Conceptualization, Data curation, Formal Analysis, Methodology, Writing – original draft. AR: Methodology, Writing – review & editing. BB: Conceptualization, Formal Analysis, Methodology, Project administration, Supervision, Writing – original draft, Writing – review & editing. DM: Methodology, Project administration, Supervision, Writing – review & editing. FrS: Conceptualization, Methodology, Project administration, Supervision, Writing – review & editing.

## References

[1] Swinburn BA Kraak VI Allender S Atkins VJ Baker PI Bogard JR. The global Syndemic of obesity, undernutrition, and climate change: the lancet commission report. Lancet. (2019) 393:791–846. doi: 10.1016/S0140-6736(18)32822-8, PMID: 30700377

[2] Willett W Rockström J Loken B Springmann M Lang T Vermeulen S. Food in the Anthropocene: the EAT–lancet commission on healthy diets from sustainable food systems. Lancet. (2019) 393:447–92. doi: 10.1016/S0140-6736(18)31788-4, PMID: 30660336

[3] Fanzo J Rudie C Sigman I Grinspoon S Benton TG Brown ME. Sustainable food systems and nutrition in the 21st century: a report from the 22nd annual Harvard nutrition obesity symposium. Am J Clin Nutr. (2022) 115:18–33. doi: 10.1093/ajcn/nqab315, PMID: 34523669 PMC8755053

[4] CFS (2021). CFS Voluntary Guidelines on Food Systems and Nutrition. Committee on World Food Security, Rome. Available at: https://www.fao.org/cfs/vgfsn/en/

[5] Fernqvist F Ekelund L. Credence and the effect on consumer liking of food—a review. Food Qual Prefer. (2014) 32:340–53. doi: 10.1016/j.foodqual.2013.10.005

[6] Marette S. Quality, market mechanisms and regulation in the food chain. Bio Appl Econ. (2016) 5:217–35. doi: 10.13128/BAE-18766

[7] Cecchini M Warin L. Impact of food labelling systems on food choices and eating behaviours: a systematic review and meta-analysis of randomized studies. Obes Rev. (2016) 17:201–10. doi: 10.1111/obr.12364, PMID: 26693944

[8] OECD. The Heavy Burden of Obesity: The Economics of Prevention. Paris, France: OECD Health Policy Studies, OECD Publishing (2019).

[9] Khandpur N Swinburn B Monteiro CA. Nutrient-based warning labels may help in the pursuit of healthy diets. Obesity (Silver Spring). (2018) 26:1670–1. doi: 10.1002/oby.22318, PMID: 30358147

[10] Golan E Kuchler F Mitchell L Greene C Jessup A. Economics of food labeling. J Consum Policy. (2001) 24:117–84. doi: 10.1023/A:1012272504846, PMID: 38458401

[11] ISO (2019). Environmental labels. Geneva, Switzerland: International Organization for Standardization.

[12] European Commission (2023). European Commission-food, farming, fisheries-food safety. EU law on food information to consumers. Available at: https://food.ec.europa.eu/safety/labelling-and-nutrition/food-information-consumers-legislation_en

[13] Yokessa M Marette S. A review of eco-labels and their economic impact. International review of. Environ Resour Econ. (2019) 13:119–63. doi: 10.1561/101.00000107

[14] Manta F Campobasso F Tarulli A Morrone D. Showcasing green: how culture influences sustainable behavior in food eco-labeling. Br Food J (2021) doi: 10.1108/BFJ-05-2021-0478 (Epub ahead of print).

[15] The Consumer Goods Forum, Institut du Commerce, ADEME (2022). Empowering Consumer Choice and Eco Design in France–Best Practices for FMCG Companies with Environmental Labelling of Food. Available at: https://www.theconsumergoodsforum.com/global-learning-mechanism-resources/empowering-consumer-choice-and-eco-design-in-france-best-practices-for-fmcg-companies-with-environmental-labelling-of-food/

[16] Nes K Antonioli F Ciaian P. Trends in sustainability claims and labels for newly introduced food products across selected European countries. Agribusiness. (2024). doi: 10.1002/agr.21894

[17] US EPA (2014). Introduction to ecolabels and standards for greener products. Available at: https://www.epa.gov/greenerproducts/introduction-ecolabels-and-standards-greener-products

[18] Torma G Thøgersen J. A systematic literature review on meta sustainability labeling—what do we (not) know? J Clean Prod. (2021) 293:126194. doi: 10.1016/j.jclepro.2021.126194

[19] Ecolabel Index Who’s deciding what’s green?. (2023). Available at: https://wwww.ecolabelindex.com (Accessed June 6, 2023).

[20] European Commission (2023). Product groups and criteria. EU ecolabel product groups and criteria. Available at: https://environment.ec.europa.eu/topics/circular-economy/eu-ecolabel/product-groups-and-criteria_en

[21] European Commission (n.d.) Step-by-step support for applying for an EU ecolabel for your goods or services. How to Apply for the EU Ecolabel Available at: https://environment.ec.europa.eu/topics/circular-economy/eu-ecolabel/how-apply_en

[22] Tranfield D Denyer D Smart P. Toward a methodology for developing evidence-informed management knowledge by means of systematic review. Br J Manag. (2003) 14:207–22. doi: 10.1111/1467-8551.00375

[23] Gutiérrez NL Valencia SR Branch TA Agnew DJ Baum JK Bianchi PL. Eco-label conveys reliable information on fish stock health to seafood consumers. PLoS One. (2012) 7:e43765. doi: 10.1371/journal.pone.0043765, PMID: 22928029 PMC3424161

[24] Katt F Meixner O. A systematic review of drivers influencing consumer willingness to pay for organic food. Trends Food Sci Technol. (2020) 100:374–88. doi: 10.1016/j.tifs.2020.04.029

[25] Marette S. Ecological and/or nutritional scores for food traffic-lights: results of an online survey conducted on pizza in France. Sustain For. (2022) 14:247. doi: 10.3390/su14010247, PMID: 38467579

[26] van der Waal NE Folkvord F Azrout R Meppelink CS. Can product information steer towards sustainable and healthy food choices? A pilot study in an online supermarket. Int J Environ Res Public Health. (2022) 19:1107. doi: 10.3390/ijerph19031107, PMID: 35162129 PMC8834331

[27] van Bussel LM Kuijsten A Mars M van’t Veer P. Consumers’ perceptions on food-related sustainability: a systematic review. J Clean Prod. (2022) 341:130904. doi: 10.1016/j.jclepro.2022.130904

[28] Teisl MF Roe B Hicks RL. Can eco-labels tune a market? Evidence from dolphin-safe labeling. J. Environ. Econ. Manage. (2002). 43, 339–359.

[29] Arrazat L Chambaron S Arvisenet G Goisbault I Charrier JC Nicklaus S. Traffic-light front-of-pack environmental labelling across food categories triggers more environmentally friendly food choices: a randomised controlled trial in virtual reality supermarket. Int J Behav Nutr Phys Act. (2023) 20:7. doi: 10.1186/s12966-023-01410-8 36703160 PMC9881283

[30] Potter C Pechey R Cook B Bateman P Stewart C Frie K. Effects of environmental impact and nutrition labelling on food purchasing: an experimental online supermarket study. Appetite. (2023) 180:106312. doi: 10.1016/j.appet.2022.106312, PMID: 36150553

[31] Carlsson F Kataria M Lampi E. Sustainable food: can information from food labels make consumers switch to meat substitutes? Ecol Econ. (2022) 201:107567. doi: 10.1016/j.ecolecon.2022.107567

[32] Duckworth JJ Randle M McGale LS Jones A Doherty B Halford JCG. Do front-of-pack “green labels” increase sustainable food choice and willingness-to-pay in UK consumers? J Clean Prod. (2022) 371:133466. doi: 10.1016/j.jclepro.2022.133466

[33] De Bauw M De La Revilla LS Poppe V Matthys C Vranken L. Digital nudges to stimulate healthy and pro-environmental food choices in E-groceries. Appetite. (2022) 172:105971. doi: 10.1016/j.appet.2022.105971, PMID: 35181380

[34] Norton V Waters C Oloyede OO Lignou S. Exploring consumers’ understanding and perception of sustainable food packaging in the UK. Food Secur. (2022) 11:3424. doi: 10.3390/foods11213424, PMID: 36360036 PMC9657940

[35] Wolfson JA Musicus AA Leung CW Gearhardt AN Falbe J. Effect of climate change impact menu labels on fast food ordering choices among US adults: a randomized clinical trial. JAMA Netw Open. (2022) 5:e2248320. doi: 10.1001/jamanetworkopen.2022.48320, PMID: 36574248 PMC9857560

[36] Tobi RCA Harris F Rana R Brown KA Quaife M Green R. Sustainable diet dimensions. Comparing consumer preference for nutrition, environmental and social responsibility food labelling: a systematic review. Sustain For. (2019) 11:6575. doi: 10.3390/su11236575 PMC7616258 39035350

[37] Kühne SJ Reijnen E Laasner Vogt L Baumgartner M. Can carbon labels encourage green food choices? Front Psychol. (2023) 13:902869. doi: 10.3389/fpsyg.2022.902869 36778167 PMC9912457

[38] Xu H Xiao M Zeng J Hao H. Green-labelled rice versus conventional rice: Perception and Emotion of Chinese Consumers Based on Review Mining Foods. Food Secur. (2023) 12:87. doi: 10.3390/foods12010087 PMC9818160 36613303

[39] Binnekamp M Ingenbleek P. Do “good” food products make others look “bad”? Br Food J. (2008) 110:843–64. doi: 10.1108/00070700810900576

[40] Camilleri AR Larrick RP Hossain S Patino-Echeverri D. Consumers underestimate the emissions associated with food but are aided by labels. Nat Clim Chang. (2019) 9:53–8. doi: 10.1038/s41558-018-0354-z

[41] Hallez L Qutteina Y Boen F Smits T. The ABC’s of ecological and nutrition labels. The impact of label theme and complexity on the environmental footprint of online grocery choices. Sustain For. (2021) 13:2474. doi: 10.3390/su13052474

[42] Neumayr L Moosauer C. How to induce sales of sustainable and organic food: the case of a traffic light eco-label in online grocery shopping. J Clean Prod. (2021) 328:129584. doi: 10.1016/j.jclepro.2021.129584

[43] Zepeda L Sirieix L Pizarro A Corderre F Rodier F. A conceptual framework for analyzing consumers’ food label preferences: an exploratory study of sustainability labels in France, Quebec, Spain and the US. Int J Consum Stud. (2013) 37, 605–616. doi: 10.1111/ijcs.12041

[44] Wakamatsu M Managi S. Does spatially targeted information boost the value of ecolabeling seafood? A choice experiment in Japan. Appl Econ. (2022) 54:6008–21. doi: 10.1080/00036846.2022.2056127

[45] Fretes G Sepúlveda A Corvalán C Cash SB. Children’s perceptions about environmental sustainability, food, and nutrition in Chile: a qualitative study. Int J Environ Res Public Health. (2021) 18:9679. doi: 10.3390/ijerph18189679, PMID: 34574616 PMC8467886

[46] Engels SV Hansmann R Scholz RW. Toward a sustainability label for food products: an analysis of experts’ and consumers’ acceptance. Ecol Food Nutr. (2010) 49:30–60. doi: 10.1080/03670240903433154, PMID: 21883088

[47] Tait P Saunders C Guenther M Rutherford P Miller S. Exploring the impacts of food label format on consumer willingness to pay for environmental sustainability: A choice experiment approach in the United Kingdom and Japan. Int Food Res J. (2016) 23:1787–96. doi: 10.5555/20163247262

[48] Grebitus C Steiner B Veeman MM. Paying for sustainability: a cross-cultural analysis of consumers’ valuations of food and non-food products labeled for carbon and water footprints. J Behav Exp Econ. (2016) 63:50–8. doi: 10.1016/j.socec.2016.05.003

[49] Abdu N Mutuku J. Willingness to pay for socially responsible products: a meta−analysis of coffee ecolabelling. Heliyon. (2021) 7:e07043. doi: 10.1016/j.heliyon.2021.e07043 34235279 PMC8246260

[50] De Valck J Rolfe J Rajapaksa D Star M. Consumers’ preferences and willingness to pay for improved environmental standards: insights from cane sugar in the great barrier reef region*. Aust J Agric Resour Econ. (2022) 66:505–31. doi: 10.1111/1467-8489.12484

[51] Ma X Liu Z Meng T Florkowski WJ Mu Y. Impact of food sustainability labels on the Price of Rice in online sales. Food Secur. (2022) 11:3781. doi: 10.3390/foods11233781, PMID: 36496589 PMC9738476

[52] Nian Y Gao Z Zhao R. Are people’s daily life habits consistent with their preference for food sustainability labels? Agribusiness. (2023) 39:589–622. doi: 10.1002/agr.21803

[53] Nydrioti I Grigoropoulou H. Using the water footprint concept for water use efficiency labelling of consumer products: the Greek experience. Environ Sci Pollut Res Int. (2023) 30:19918–30. doi: 10.1007/s11356-022-23573-w, PMID: 36242669 PMC9938042

[54] Sörqvist P Hedblom D Holmgren M Haga A Langeborg L Nöstl A. Who needs cream and sugar when there is eco-labeling? Taste and willingness to pay for “eco-friendly” coffee. PLoS One. (2013) 8:e80719. doi: 10.1371/journal.pone.0080719, PMID: 24324623 PMC3851458

[55] Lusk JL Jamal M Kurlander L Roucan M Taulman L. A meta-analysis of genetically modified food valuation studies. J Agric Resour Econ. (2005) 30:28–44.

[56] Bastounis A Buckell J Hartmann-Boyce J Cook B King S Potter C. The impact of environmental sustainability labels on willingness-to-pay for foods: a systematic review and Meta-analysis of discrete choice experiments. Nutrients. (2021) 13:2677. doi: 10.3390/nu13082677, PMID: 34444837 PMC8398923

[57] Aprile MC Punzo G. How environmental sustainability labels affect food choices: assessing consumer preferences in southern Italy. J Clean Prod. (2022) 332:130046. doi: 10.1016/j.jclepro.2021.130046

[58] Zhu Z Zhang T Hu W. The accumulation and substitution effects of multi-nation certified organic and protected eco-origin food labels in China. Ecol Econ. (2023) 203:107625. doi: 10.1016/j.ecolecon.2022.107625

[59] Sigurdsson V Larsen NM Pálsdóttir RG Folwarczny M Menon RGV Fagerstrøm A. Increasing the effectiveness of ecological food signaling: comparing sustainability tags with eco-labels. J Bus Res. (2022) 139:1099–110. doi: 10.1016/j.jbusres.2021.10.052

[60] Asioli D Aschemann-Witzel J Nayga RM. Sustainability-related food labels. Ann Rev Resour Econ. (2020) 12:171–85. doi: 10.1146/annurev-resource-100518-094103, PMID: 36010389

[61] Praneetvatakul S Vijitsrikamol K Schreinemachers P. Ecolabeling to improve product quality and reduce environmental impact: a choice experiment with vegetable farmers in Thailand. Front Sustain Food Syst. (2022) 5:704233. doi: 10.3389/fsufs.2021.704233

[62] Leach AM Emery KA Gephart J Davis KF Erisman JW Leip A. Environmental impact food labels combining carbon, nitrogen, and water footprints. Food Policy. (2016) 61:213–23. doi: 10.1016/j.foodpol.2016.03.006

[63] Jaďuďová J Tomaškin J Ševčíková J Andráš P Drimal M. The importance of environmental food quality labels for regional producers: a Slovak case study. Food Secur. (2022) 11:1013. doi: 10.3390/foods11071013, PMID: 35407100 PMC8997742

[64] Gröfke N Duplat V Wickert C Tjemkes B. A multi-stakeholder perspective on food labelling for environmental sustainability: attitudes, perceived barriers, and solution approaches towards the “traffic light index”. Sustain For. (2021) 13:933. doi: 10.3390/su13020933

[65] Bunge AC Wickramasinghe K Renzella J Clark M Rayner M Rippin H. Sustainable food profiling models to inform the development of food labels that account for nutrition and the environment: a systematic review. Lancet Planet Health. (2021) 5:e818–26. doi: 10.1016/S2542-5196(21)00231-X, PMID: 34774122

[66] Chang MY Chen HS. Understanding consumers’ intentions to purchase clean label products: evidence from Taiwan. Nutrients. (2022) 14:3684. doi: 10.3390/nu14183684 36145062 PMC9503815

[67] Sonntag IW Lemken D Spiller A Schulze M. Welcome to the (label) jungle? Analyzing how consumers deal with intra-sustainability label trade-offs on food. Food Qual Prefer. (2023) 104:104746. doi: 10.1016/j.foodqual.2022.104746

[68] Kaczorowska J Rejman K Halicka E Szczebyło A Górska-Warsewicz H. Impact of food sustainability labels on the perceived product value and Price expectations of urban consumers. Sustain For. (2019) 11:7240. doi: 10.3390/su11247240

[69] Daniuseviciute-Brazaite L. Education for sustainable development: sustainability-related food labels. Sustain For. (2021) 13:8117. doi: 10.3390/su13158117, PMID: 36010389

[70] Lin W Nayga RM. Green identity labeling, environmental information, and pro-environmental food choices. Food Policy. (2022) 106:102187. doi: 10.1016/j.foodpol.2021.102187

[71] Grunert KG Hieke S Wills J. Sustainability labels on food products: consumer motivation, understanding and use. Food Policy. (2014) 44:177–89. doi: 10.1016/j.foodpol.2013.12.001, PMID: 36503306

[72] Potter C Pechey R Clark M Frie K Bateman PA Cook B. Effects of environmental impact labels on the sustainability of food purchases: two randomised controlled trials in an experimental online supermarket. PLoS One. (2022) 17:e0272800. doi: 10.1371/journal.pone.0272800, PMID: 36327277 PMC9632881

[73] Muller L Lacroix A Ruffieux B. Environmental labelling and consumption changes: a food choice experiment. Environ Resour Econ. (2019) 73:871–97. doi: 10.1007/s10640-019-00328-9, PMID: 37569210

[74] Potter C Bastounis A Hartmann-Boyce J Stewart C Frie K Tudor K. The effects of environmental sustainability labels on selection, purchase, and consumption of food and drink products: a systematic review. Environ Behav. (2021) 53:891–925. doi: 10.1177/0013916521995473, PMID: 34456340 PMC8384304

[75] Schuldt JP. Does green mean healthy? Nutrition label color affects perceptions of healthfulness. Health Commun. (2013) 28:814–21. doi: 10.1080/10410236.2012.725270, PMID: 23444895

[76] Annunziata A Mariani A Vecchio R. Effectiveness of sustainability labels in guiding food choices: analysis of visibility and understanding among young adults. Sustain Product Consump. (2019) 17:108–15. doi: 10.1016/j.spc.2018.09.005

[77] Sirieix L Delanchy M Remaud H Zepeda L Gurviez P. Consumers’ perceptions of individual and combined sustainable food labels: a UK pilot investigation. Int J Consum Stud. (2013) 37:143–51. doi: 10.1111/j.1470-6431.2012.01109.x

[78] Futtrup R Tsalis G Pedersen S Dean M Benson T Aschemann-Witzel J. Is the whole more than the sum of its parts? Challenges and opportunities for a holistic consumer-friendly sustainability label on food. Sustain Product Consump. (2021) 28:1411–21. doi: 10.1016/j.spc.2021.08.014

[79] Chirilli C Molino M Torri L. Consumers’ awareness, behavior and expectations for food packaging environmental sustainability: influence of socio-demographic characteristics. Food Secur. (2022) 11:2388. doi: 10.3390/foods11162388, PMID: 36010389 PMC9407116

[80] Pechey R Bateman PA Cook B Potter C Clark M Stewart C. Testing the effectiveness of ecolabels to reduce the environmental impact of food purchases in worksite cafeterias: a randomised controlled trial. Appetite. (2022) 179:106277. doi: 10.1016/j.appet.2022.106277, PMID: 35995306 PMC7618413

