# Supplementary material for: Strengths and weaknesses of food eco-labeling: a review
Source: Front Nutr. 2024 Mar 27;11:1381135. doi: 10.3389/fnut.2024.1381135 (PMC11005915; doi:10.3389/fnut.2024.1381135)
Supplement: Supplementary file 1 [file Table_1.docx]

**Supplementary material**

**Table S1.** List of papers included in the review grouped by areas of impact or concern.

| **Reference** | **Type of study and study design/methods** | **Eco-label content/message/image** | **Main outcomes** |
| --- | --- | --- | --- |
| Change in consumer’s perception, intention or behavior | | | |
| Arrazat et al, 2023 | - Research article - 2-arm randomized controlled trial in a virtual reality supermarket (n=132) in France. | - Evaluative labels applied to food referred to the environmental footprint calculated as single score from 16 indicators. Labels included letters (A to E) and colors (green to red)   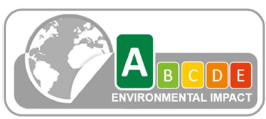 | - Sustainable food choices of single products and meals increased, while the less sustainable ones decreased. |
| Camilleri et al, 2019 | - Research article - Modelling study based on online surveys carried out virtually | - Evaluative and descriptive carbon footprint label applied food: black lightbulb with quantitative data and a colored score below (green to red), plus text. | - Ecolabels such as light-bulb minutes shift purchase choice towards lower emission options. |
| Carlsson et al, 2022 | - Research article - Online survey with choice preferences in Sweden (n=1471) | - Evaluative and descriptive ecolabels (n=3) applied to food: climate impact of production (n=1); antibiotics use and animal care (n=1); one health label (n=1). - All labels had 3 levels of impact and used traffic light colors.   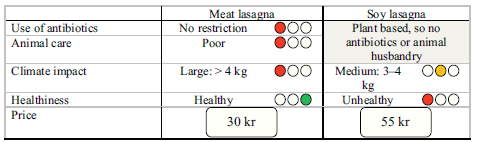 | - The ecolabel was first explained to consumers (e.g., what antibiotic use is); 25% chose consistently the ecolabelled meat or meat alternative, 75% are willing to choose the ecolabel versions but under certain conditions. |
| De Bauw et al, 2022 | - Research article - Two-stage randomized controlled trial on household food decision makers experiment for an e-grocery (n=994) in Belgium | - Evaluative labels for food: Eco-score and Nutri-score. | - Displaying a combined Nutri- and Eco-Score at product level improved the nutritional quality of selected food baskets. - Nutri- and Eco-Score led to shifting behaviour in environmental impact when facilitated with recommendation agents. - The display of the average impact scores of the selected basket and of social norms did not lead to additional improvements. |
| Duckworth et al, 2022 | - Research article - Two online surveys containing a hypothetical online shopping experience on “meat eater” consumers (Exp 1, n=875; Exp 2, n=1080) in UK | - Evaluative labels applied to food, presenting blue globes with text (sustainably sourced, locally sourced, environmentally friendly or low GHGE).   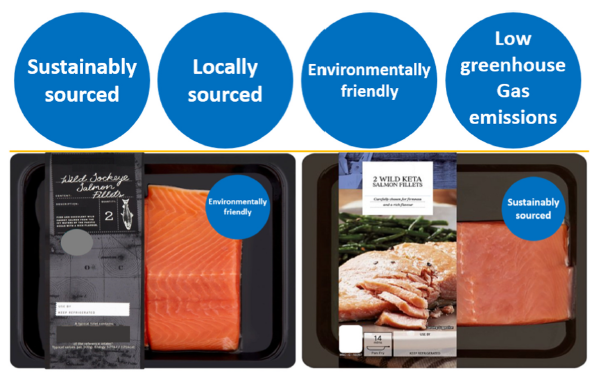 | - Consumers choose more and were willing-to-pay more for green labelled products. - ‘Sustainably sourced’ and ‘Locally sourced’ products were chosen most with the highest WTP for ‘Locally sourced’ products. - Environmental concern consistently predicted preference for labelled products. |
| Fretes et al, 2021 | - Research article - Online focus groups (n=8) in Chile in children (n=30) | - Evaluative invented labels (n=4) not specified if for food or package: Graphic colored content with text; graphic with text and colors (green to red); QR code; animal welfare, eco-friendly and carbon footprint graphics with text and color (green).   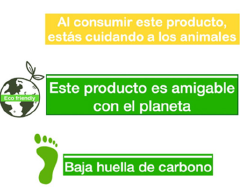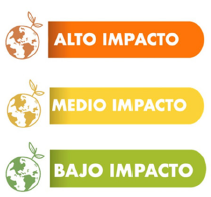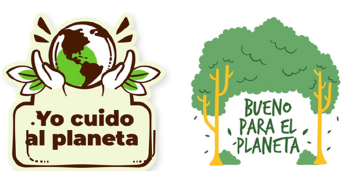 | - Environmental sustainability was not a familiar concept. - Participants showed awareness about the environmental impact of their eating behavior. - Children had a positive perception of eco-labels. |
| Kühne et al, 2023 | - Research article - Online survey (n=402) in Switzerland | - Evaluative and evaluative and descriptive carbon labels for food: traffic light label, green foot, and CO_2_ star rating.   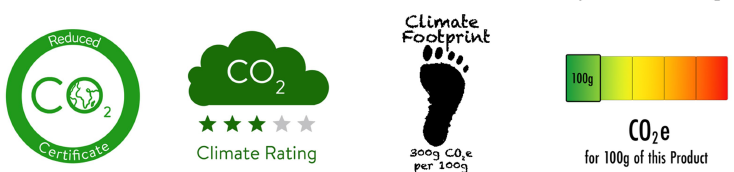 | - Label presence increased preference, which was highest for the traffic light one. |
| Norton et al, 2022 | - Research article - Online survey (n=405) in UK | - No logo used, only text in the packaging using ready to eat cooked items and packaging ranking with materials recycling information.   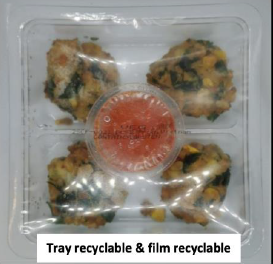 | - Labels encouraged more sustainable purchases, even more important than shelf life and use-by-date. |
| Potter et al, 2023 | - Research article - Randomized control trial applying an experimental online supermarket platform (participants, n=2730) in UK | - Colored (green to red) globes with letters (A to E) with title (environmental score), plus the health score above (scale, same colors and letters). - Evaluative labels for food.   ~~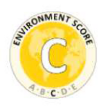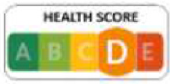~~ | - Products with labels lowered the environmental impact score of the purchase. - There results did not vary if a nutritional label was present. |
| Teisl et al, 2002 | - Research article | - Dolphin-safe label. - Evaluative label (not shown). | - Dolphin-safe ecolabel increased sales of canned tuna. - Consumers may not respond instantaneously. |
| Tobi, 2019 | - Systematic review (articles, n=30; participants, n=19040) | - Focused on label preferences (nutrition, social or environmental) by consumers and WTP. | - Consumers value environmental and social attributes more than nutrition information (57% of studies) - Organic is the most preferred ecolabel but also the most frequently tested in comparison to ecological claims, carbon emissions, and water footprint labels. |
| Van Bussel, 2022 | - Systematic review (quantitative studies, n=76; quantitative studies, n=49; qualitative studies, n=21) | - Label content was divided into different domains (e.g., product, transportation, etc.). | - Production (31%), transportation (19%) and product (14%) were the largest domains identified in quantitative studies; production (25%), consumer and product (20% each), were the main identified in qualitative ones. - Consumers are not well aware of food-related sustainability topics, as they believe it does not influence their food choices. - Price, taste and individual health are more influential than sustainability for consumers. |
| Wakamatsu & Managi, 2022 | - Research article - Online survey (n=3596) in Japan with choice experiment | - Ecolabel of sustainable harvesting without known certification (e.g. MSC) but with third party certification. | - Knowledge did not seem to influence the value of ecolabels. - Ecolabels generate premium prices, more so if world fishery information is provided. |
| Wolfson et al, 2022 | - Research article - Randomized controlled trial - Online survey in USA (n=5049) | - Evaluative and descriptive label (text) for food. - 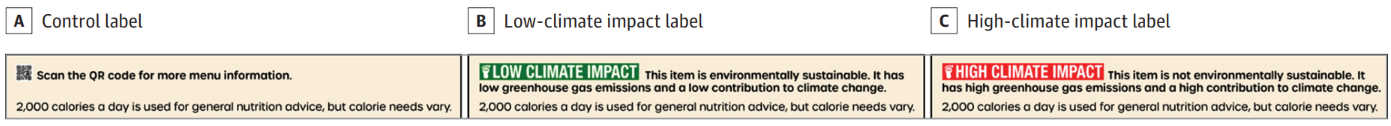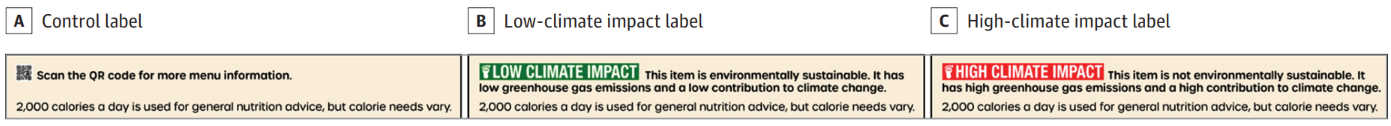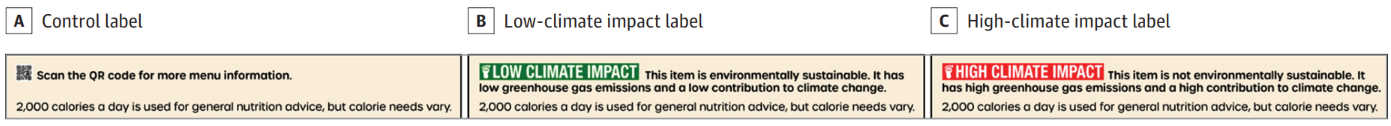 | - Eco-labels, especially the negative ones, encourages more sustainable options. |
| Xu et al, 2023 | - Research article - Used JD.com to obtain consumers’ review data using text mining (comments, n=82398) - Compared conventional rice vs green-labelled rice in China | - Evaluative green logo with text (GreenFood) for food.   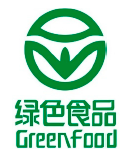 | - Consumers were more satisfied with green-labelled rice and higher perceived quality. - Mistrust of green labels is one of the main causes of negative emotions. |
| Zepeda et al, 2013 | - Research article - Online survey in France, Canada, Spain and US (participants, n=375) | - Used 6 pairs of labels randomly generated from 16 actual labels from each country (organic, origin, fair trade, nutrition and environmental). - Evaluative labels for food. | - Differences between nations are substantial, as well as their reasons for choosing foods. - Reasons include label’s message, design and source, and the consumers’ values. |
| Price and value relationship between food products and eco-labelling | | | |
| Abdu & Motuku, 2021 | - Meta-analysis (studies, n=22; observations, n=97) | - Organic, COOL, ST10, Fairtrade and Rainforest, among others | - Consumers WTP for ecolabels coffee is influenced by the label. - Organic labels influence the most coffee consumers WTP. - There is a regional difference in the WTP for ecolabelled coffee products. |
| Aprile & Punzo, 2022 | - Research article - In-presence survey in Italy (participants, n=192) | - Food labels of origin of the product, Rainfores Allianca Certified, EU organic, and “per il clima-legambiente”. - Evaluative (n=2) and descriptive (n=1) labels. | - Higher understanding of the ecolabel increases sustainable product preferences. - Higher educational level increases relevance of ecolabels. - WTP increases with ecolabels with higher market penetration rather than third party certifications. |
| Bastounis et al, 2021 | - Systematic review and meta-analysis (studies, n=35; participants, n=35725) - Discrete choice experiments on willingness to pay | - Organic; sustainable (e.g., different levels of GHG emissions reduction or water efficiency); GHG emissions; water management; biodiversity. | - A mean willingness of 3.79 PPP$/kg for ecolabelled foods was found. - Organic labels showed higher WTP. - Women and less educated people expressed higher WTP. |
| De Valck et al, 2022 | - Research article - Online survey in Australia (n=1100) | - Great Barrier Reef (GBR)-safe ecolabel, not shown. | - Health perception is more important than sustainability in sugar buying decisions. - Consumers are willing to pay a premium (0.34%) for ecolabelled products |
| Katt & Meixner, 2016 | - Systematic review (articles, n=138) - WTP for organic food | - Organic labels | - Higher WTP for organic labels. |
| Lusk et al, 2005 | - Research article - Meta-analysis | - GM label, not shown | - The product itself affect valuations. - Nutritionally rich GM food decreased premiums for non-GM food. |
| Ma et al, 2022 | - Research article - Online survey to collect e-commerce data of type of rice, labels and price, among other characteristics (n=2492) in China | - Existing 3 labels with logo, colors (mostly green) and text. - Labels referred to green food and organic food. - Evaluative labels for food.    | - Labelled rice showed a willing to pay a higher price as a premium (47.5%), and differences among labels were found (higher price for organic than for the green label). |
| Nian et al, 2023 | - Research article - Online survey (n=667) in US and University focus groups | - 4 labels: USDA organic, non-GMO, ecofriendly and fair trade - Evaluative labels for food. | - Sustainable products were perceived as healthier. - Willingness to pay was higher for labelled milk (23-36%), for strawberries (24-31%). - Differences were found with the same label but different products. |
| Sörqvist et al, 2022 | - Research article - Experiment choice (taste) - 2 experiments: N=44 and N=87 in Sweden | - Eco-label, not shown. | - Consumers preferred (taste and WTP) the eco-friendly choice (even though it was the same product). - This was not a consequence of social desirability, as it was anonymous. |
| Tait et al, 2016 | - Research article - Experiment (choice experiment) to Japanese and UK | - 3 formats, one only including text, other with graphic with text and colors and the third one is a compass also with text and colors. - Evaluative labels for food and packaging. | - Differences in ecolabeling format does not lead to changes in WTP. - Preferences for higher nutrition value content products are sensitive to label format, but not for carbon footprint. |
| Zhu et al, 2022 | - Research article - Choice experiment in China | - Existing organic labels. Comparison between Chinese organic label and multi-nation organic labels. - All labels consisted in logo, text and colors, but the EU organic. - Evaluative labels for food. | - WTP for milk increased for double or triple labelled products, but not for the Chinese organic label alone, but with a marginal increase in WTP for additional organic labels. |
| Producer’s perspective | | | |
| Asioli, 2020 | - Review article (n=not specified) | - Not specified | - Ecolabels impact the manufacturing process as well as food choices. - An ecolabel that involves a more integrated sustainability measure should be developed. |
| Jaďuďová et al, 2022 | - Research article - Online survey on producers from the Slovak Republic (n=39) | - 3 labels concerning local producers. All have logos but the third one that was created by the authors (“regional product”). - Evaluative labels for food. - Colors mainly red, white and blue with text.   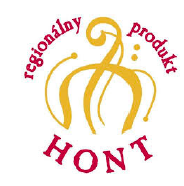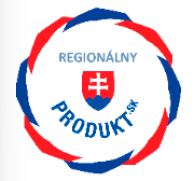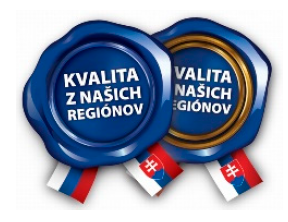 | - Most of the producers (72%) are aware of the concept of a regional product and associate it (82%) with tradition and a specific region. - A subgroup (36%) considers regional product labelling to be a tool to support the development of tourism and joined this scheme (26%) to add value to their products and help make consumers perceive them as safer products of higher quality. - Joining the scheme did not bring higher profits to the producers. |
| Leach et al, 2016 | - Research article - Estimation methods used data from the US | - Evaluative and descriptive carbon, nitrogen, and water footprints content for food. - Graphic consisted in stars label, stoplight label, nutrition label add-on, and a detailed comparison label. | - Different estimation methods for environmental footprint and designs are proposed. - Authors suggest having a multiple and integrated environmental impact label. |
| Praneetvatakul et al, 2022 | - Research article - Farmer’s survey in Thailand (n=303) and choice experiment | - Labels with logo, colors and text. - Evaluative labels for food.   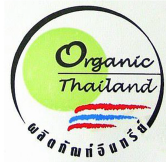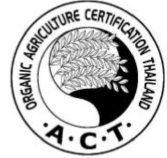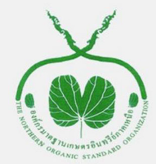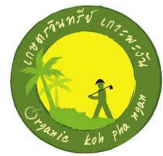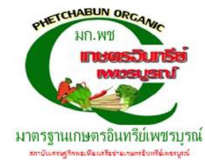 | - Farmers prefer having an ecolabel. - For farmers, long-term health effects and training were the most important aspects, while supermarket and export were not significant. - Farmers have a marginal willingness to pay for ecolabels, and lower prices for riskier to pests’ crops. |
| Institutional framework and label development | | | |
| Bunge et al, 2021 | - Review article (included studies, n=10) | - Focuses on sustainable food profiling models (SFPM). | - Few SFPM include more than one indicator, even fewer include nutritional values or other dimensions of sustainability. - There is a need for consistency and to develop reference values. |
| Chang & Chen, 2022 | - Research article - Online survey (n=265) in Taiwan | - “Clean label” described for a simple process, avoidance of unnecessary food additives and palatability. - Evaluative label for food. | - Consumers were influenced by their involvements with the label, as well as their knowledge of the label - Trust is key to purchase intention |
| Daniuseviciute-Brazaite, 2021 | - Research article - Online survey in Lithuania (participants, n=384) | - Lithuanian national quality product (NQP) label, which stands for the nutritional content, natural and environmentally friendly and ideally locally sourced. - Evaluative label for food. | - Less than half of the respondents knew the Lithuanian integrated label (include environment, nutrition and naturalness), therefore misunderstand it as they considered it was not sufficiently clear. - Higher education level increase purchase of products with the label. |
| Gröfke et al, 2021 | - Research article - Exploratory interviews in Netherlands, Germany and Belgium (participants, n= 16) | - Stoplight labels in general. - Evaluative labels focused on food   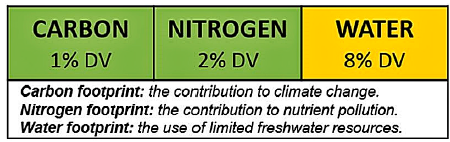  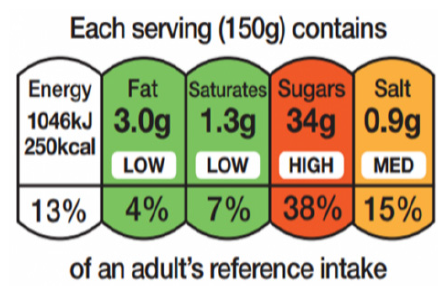 | - Food labelling should have a holistic approach. - Interaction among stakeholders affects the implementation of ecolabels and could even inhibit it. |
| Grunert et al, 2014 | - Research article - Online survey in Europe (participants, n=4408) | - Fair Trade, Animal Welfare, Rainforest Alliance, Carbon Footprint, EU eco-label. - Evaluative (n=4) and descriptive label (n=1) for food.    | - Self-explanatory labels reach higher understanding. - Country differences, human values and demographic characteristics impact understanding and use of sustainability labels. - Low level of use of sustainability labels in general. |
| Gutiérrez et al, 2012 | - Research article - Modelling study | - Marine Stewardship Council (MSC) ecolabel. - Evaluative label for food. | - Sustainable fishing certifications improve stocks. - Certified fisheries were above biomass levels that would produce maximum sustainable yield, meanwhile non-certified fisheries reach lower levels. - Certified stocks had much lower mean exploitation rates compared for those that did not want the certification. |
| Kaczorowska et al, 2019 | - Research article - Online survey in Poland - (participants n=423) | - EU organic, Protected geographical indication (PGI) or Fairtrade. - Evaluative labels for food. | - Unfamiliar logos prevent costumers (even those sensible to environmentally friendly products) from using them. - Urban consumers are not willing to pay higher prices for ecolabelled foods. |
| Lin & Nayga, 2022 | - Research article - Online choice experiment in US (participants, n=1034) | - USA organic, Fairtrade, Carbon trust label. - Evaluative labels for food. | - Providing sustainability information and having eco-labels increases valuation of already existing sustainable products. - Ecolabels by their own did not reach these results. |
| Eco-label design and excessive information | | | |
| Annunziata et al, 2019 | Research article  - Online survey (participants, n=305) in Italy | - EU organic, Fairtrade, Rainforest Alliance certified and Libera Terra. - Evaluative labels for food. | - Visibility of ecolabels are low: up to 75% did not notice the label. - The understanding of the label is low, but for the organic label: only 15% understood the ecolabel. - Higher visibility leads to higher understanding. Therefore, authors suggest ecolabels should be coupled with education public policies. |
| Chirilli et al, 2022 | - Research article  Online survey on adults (n=646) in Italy | - Green Dot, universal recycling symbol, resin identification codes, Seedling® compostable label, Forest Stewardship Council (FSC) label, Cradle to cradle® certification label, pitch-in symbol/do not litter. - Evaluative label for packaging. | - Gender, age, and education level differently affected consumer awareness, behavior, and expectations in relation to the environmental sustainability aspects of food packaging - The most sustainable groups were mainly composed of females, while less sustainable consumers were mainly the youngest. |
| Futtrup et al, 2021 | - Research article - European experts' interviews (participants, n=24) | - Not specified. | - Eco-label effectiveness could be improved by providing information that allows comparations. - Application should be mandatory to all products. - Eco-label should be coupled within a larger policy set. |
| Marette, 2022 | - Research article - Online survey (n=1200) in France | - Nutri-Score (scale from A-E and green-red) and Eco-Score, which was a leaf that had letters (A-E) and colors (green-red), but not shown within a scale. | - Labels affected purchase intention, mostly shown regarding the avoidance of the red labels. |
| Muller et al, 2019 | - Research article - Online choice experiment in France (participants, n=275) | - 3 formats, all including title. One with a car inside with a value, other a colored (red, yellow or green) globe, other with table with text and colored globes. - Evaluative (n=2) and descriptive (n=1) labels for food. | - Front of package eco-labels improve the environmental sustainability of the food basket. - Nutritional labels and eco-labels in the same product induce trade-offs. - Simpler labels are more effective. |
| Potter et al, 2021 | - Systematic review and metanalysis (studies, n=56; participants, n=42768) | - Variety of formats and messages - Ecolabels of social responsibility, animal welfare or genetic modification were excluded. | - Ecolabels are presented most as text, followed by a combination of logo and text, and, less commonly, as logo only. - Ecolabeled products with messages are more purchased. |
| Potter et al, 2022 | - Research article - Two randomized controlled trials in UK (n=1051; n=4979) - Experimental online supermarket | Different logos for food were used:   - Evaluative labels (n=4):   - scale logo with title. Colors from green to red and with letters (A-E);  - scale in letters (A-E) and colors (green-red);  - only colored circles with letters (A-E);  - circles (green to red) with text (“better” or “worse”).   - Evaluative and descriptive (n=2):   - petals with colors (green to red);  - petal size and letters (A-E).  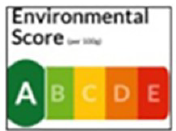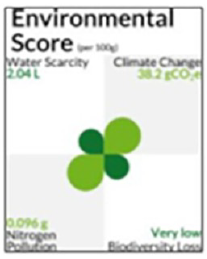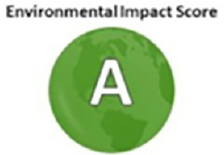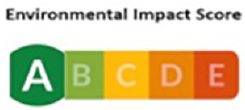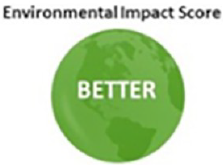 | - Green globes ecolabel was not effective in reducing environmental impact, purchases whereas colored globes with letters (A to E) and red globes labels were. |
| Sirieix et al, 2013 | - Research article - Focus groups (n=16) in the UK | - 13 ecolabels (Fairtrade, Carbon Trust, Climate friendly food, organize farmers and growers, EU organic, Nurture Tesco, Leaf, COGS, BioEquitable, Made in, Quality and origin), and nutritional information labels. - Evaluative (n=10) and descriptive (n=1) ecolabels for food. | - More labels do not necessarily mean higher value for consumers, unless they are easy to understand. - Consumers tend to look for labels that have value to them. - Organic and fair-trade labels are positively valued, but unfamiliar labels and vague terms are not. |
| **Mixed areas of impacts/concerns** | | | |
| Binnekamp & Ingenbleek, 2008 *(Cited in the following sections: change in consumers’ perception, intention or behavior; producers’ perspective)* | - Research article - Theoretical food purchasing scenario (5 in total, of which n=39, 80, 20, 85, 39) + focus group (2, of which n=6, 7) in Netherlands | - Animal welfare label and Utz Kapeh fair trade label. - Label design not shown. | - Consumers do not negatively perceive brands in the presence of an ecolabel. - A spin-off effect is unlikely by the presence of ecolabels. |
| Engels et al, 2010 *(Cited in the following sections: change in consumers’ perception, intention or behavior; institutional framework and label development)* | - Research article - Expert interviews (n=65) and online consumer survey (participants, n=233) in Switzerland | - Evaluative labels applied to food, with graphics, text, and colors. - Framework considered within the label: production method, origin, packaging, conservation, and consumption. |  |
| Golan et al, 2001 *(Cited in the following sections: price and value relationship between food products and eco-labelling; producers’ perspective; institutional framework* *and label development)* | - Research article - Mainly focused on US | - Case studies based on dolphin safe label, nutrition labeling, and organic label. - Evaluative labels for food. | - Labels are included if costs of implementation are less than the revenues. - Mandatory labelling has to be accompanied with clear standards, testing and certification services and enforcement. If not, it is more probable to disrupt the market and avoid confusion. - Importance of different labels could confuse consumers. - Labelling implementation could lead to higher prices for consumers. Thus, a redistribution of welfare from low-income to high-income households could occur. |
| Grebitus et al, 2016 *(Cited in the following sections: price and value relationship between food products and eco-labelling; institutional framework* *and label development)* | - Research article - Surveys (choice experiment) to Canadians and Germans (participants, n=3130) | - Descriptive footprint labels in text (carbon and water) for food and non-food items. | - Products with higher footprints must have an interesting discount for consumers to buy them. - High importance of eco-labelling regardless of culture, even though Germans tend to prefer more sustainable products. |
| Hallez et al, 2021 *(Cited in the following sections: change in consumers’ perception, intention or behavior; eco-label design* *and excessive information)* | - Research article - Two online choice experiment (n=142; 250) in Belgium | - Evaluative Eco-score label with or without Nutri-score label applied to food, with letters (A to E) and colors (green to red).   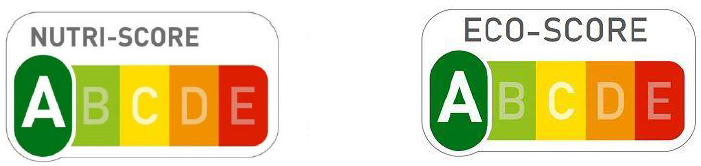 | - Young consumers are influenced by the presence of an ecolabel when purchasing. The final meals become more sustainable. - The complexity of the label did not influence the outcome. |
| Manta et al, 2021 *(Cited in the following sections: price and value relationship between food products and eco-labelling; institutional framework* *and label development)* | - Research article - Econometric modelling | - 148 labels considered, 44 countries. | - Ecolabels are more present in wealthier countries. |
| Neumayr & Moosauer, 2021 *(Cited in the following sections: change in consumers’ perception,* *intention or behavior; eco-label design* *and excessive information)* | - Research article - Online survey + online experiment in Germany (participants, n=388). | - Evaluative and Evaluative and descriptive labels for food: colored-graphic (green to red) with letters (A to G); Black text table with indicators numbers; Globes with letters and colors and a lightbulb above with text; Black foot with text within; simple Eco-Score (color, letters and title). | - Ecolabels have a significant effect, even more when a traffic-light is shown in comparison to monochromatic ones. - Greatest reductions in less sustainable products. |
| Nydrioti & Grigoropoulou, 2023 *(Cited in the following sections: price and value relationship between food products and eco-labelling; eco-label design* *and excessive information)* | - Research article - Online survey in Greece (n=326) | - No logo nor label was used, but perceptions asked according to different statements. | - Unawareness of the content of the label (26%) and preference (79%) for the description of the amount of water used for production rather than stating the company water policy, although less than half of participants know what the water footprint is. - 71% are willing to pay 5% more for a product produced with lower water, and also prefer companies that are environmentally aware. |
| Sigurdsson et al, 2022 *(Cited in the following sections: price and value* *relationship between food products and eco-labelling; eco-label design* *and excessive information)* | - Research article - Online choice experiment in the US (participants, n=1201) | - Marine Stewardship Council (MSC) and Aquaculture Stewardship and the Council (ASC) label. - Evaluative labels for food. | - Sustainability tags (images, phrases or words such as “local”, or “eco-friendly”) were preferred over ecolabels (third-party certifications). - WTP is higher if the label is familiar, up to 23% more. |
| Sonntag et al, 2023 *(Cited in the following sections: institutional framework* *and label development; eco-label design* *and excessive information)* | - Research article - Online questionnaire (n=985) in Germany and an online choice experiment for WTP | - Nutri-score, German animal welfare ecolabel “Haltungsform”, German organic label, CO_2_ “eco-score” (similar to utria-score). - Evaluative labels for food.   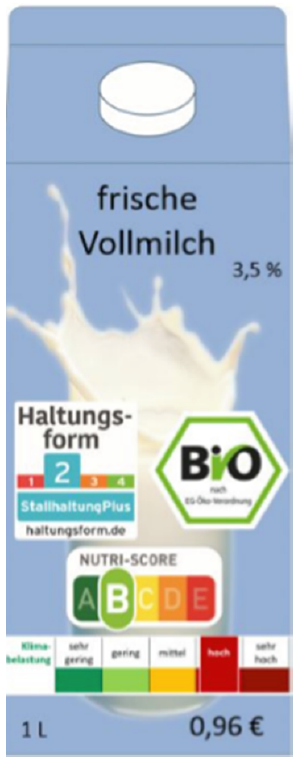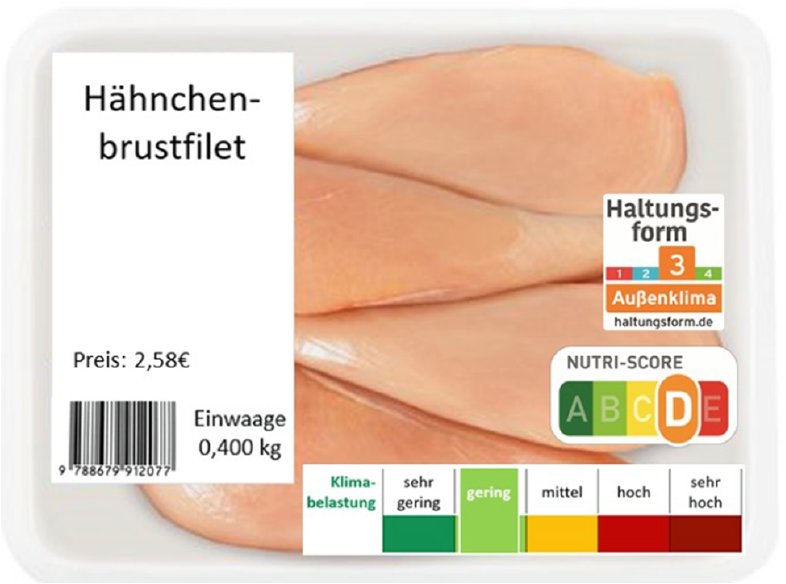 | - Participants preferred multi-level labels (e.g. nutrition + environmental label). - Organic label was not the preferred ecolabel, but other ecolabels were preferred, as organic was “not new/nothing special”. - Participants showed a very high level of awareness but very low level of trust. - WTP was highest for Nutri-score, followed by ecolabels. Both had higher WTP compared to non-labelled items. |
| Van der Waal et al, 2022 *(Cited in the following sections: change in consumers’ perception,* *intention or behavior; eco-label design* *and excessive information)* | - Research article - Pilot study in an online supermarket applying one-factor design with 3 experimental conditions on adults (n=101) in the Netherlands | - Sustainability claims (e.g. sustainable choice) with or without explanations (e.g. “Seasonal veggie! This spinach is produced in the Netherlands, so no unnecessary CO_2_ emissions due to transportation” veggie and with or without health claim. - Evaluative labels for food. | - Explanatory sustainability claim led to fewer sustainable purchases through perceived consumer effectiveness only for those with low environmental attitudes. - No effects were found for the addition of a health claim. |

Note: WTP: willingness to pay; GHG: greenhouse gas. The paper from Sirieix et al, 2013 is shown as singular item but it is also included in: Tobi et al, 2019, van Bussel et al, 2022, and Asioli et al, 2020. The paper from Zepeda et al, 2013 is shown as singular item but it is also included in Tobi et al, 2019. The paper from Tait et al, 2016 is shown as singular item but it is also included in Tobi et al, 2019 and Bastounis et al, 2021. The paper from Annunziata et al, 2019 is shown as singular item but it is also included in van Bussel et al, 2022 and Asioli et al, 2020. The paper from Grunert et al, 2014 is shown as singular item but it is also included in van Bussel et al, 2022, Asioli et al, 2020 and Potter et al, 2021. The paper from Grebitus et al, 2016 is shown as singular item but it is also included in Bastounis et al, 2021. The paper from Engels et al, 2010 is shown as singular item but it is also included in Asioli et al, 2020. The paper from Miranda-Ackermann et al, 2017 is shown as singular item but it is also included in Asioli et al, 2020. The paper from Leach et al, 2016 is shown as singular item but it is also included in Bunge et al, 2021.
